# Supplementary material for: Activation of Non-Canonical Autophagic Pathway through Inhibition of Non-Integrin Laminin Receptor in Neuronal Cells
Source: Cells. 2022 Jan 29;11(3):466. doi: 10.3390/cells11030466 (PMC8833926; doi:10.3390/cells11030466)
Supplement: Supplementary file 1 [file cells-11-00466-s001.zip › cells-1431519-supplementary.pdf]

Supplementary Material

Activation of Non-Canonical Autophagic Pathway through Inhibition of Non-Integrin Laminin Receptor in Neuronal Cells

Adriana Limone [1], Iolanda Veneruso [1,2], Antonella Izzo [1], Maurizio Renna [1], Raffaella Bonavita [1], Silvia Piscitelli [1], Gaetano Calì [3], Sergio De Nicola [4,5], Patrizia Riccio [1], Valeria D'Argenio [2,6], Antonio Lavecchia [7] and Daniela Sarnataro [1]

Table S1. Full list of the primers used for qPCR analysis.

Gene	Strand	Primer's Sequence (5'->3')	Tm	GC%	Primer's Lenght (bp)	Amplicon's Size
BECN1	FW	TGAATGTCAGAACTACAAACGC	57.27	40.91	22	229
	RW	ATACTCCCGCTGGTACTGAG	58.31	55.00	20	
ULK1	FW	TGTGCCCTCATATCCAAGCT	58.78	50.00	20	240
	RW	TCAGCAACTAGATCACCTGGA	58.18	47.62	21	
PIK3C3	FW	GCAGTTCATCCAGTCGGTTC	58.92	55.00	20	157
	RW	CACACAGTATCCAGCACAGC	58.92	55.00	20	
DNM2	FW	CTCATTCTTGCCGTCACACC	59.20	55.00	20	196
	RW	CCACGCCGATATAGCCTCTT	59.40	55.00	20	
PROM2	FW	GCTTCCTTGTGCAGATCCAG	58.91	55.00	20	286
	RW	GGCAGCTCTCCTTTTAGACG	58.35	55.00	20	
APPL1	FW	ATCCGCAGACCCAAGTTACA	59.02	50.00	20	243
	RW	CTGATGCCCTACGATCCAGT	58.96	55.00	20	
SNX33	FW	CAAGCACGACCTCTTCCAAA	58.41	50.00	20	219
	RW	AAATGGTTCATCTCGGCCTG	58.24	50.00	20	
ARF1	FW	GGCGAAATTGTGACCACCAT	59.11	50.00	20	179
	RW	CGCTCTCTGTCATTGCTGTC	59.00	55.00	20	
VPS11	FW	TCCAGGCCTACAAACTACGG	59.10	55.00	20	249
	RW	TGCCATCTGTGAACCCAATG	58.45	50.00	20	
VPS18	FW	CTCGTGGTCTCCTGCAATCA	59.75	55.00	20	163
	RW	CCAGCAGATGAGAGCCAGTA	58.88	55.00	20	
CTSB	FW	AACCTTTGATGCACGGGAAC	59.04	50.00	20	217
	RW	ATAGCCACCATTACAGCCGT	59.16	50.00	20	
GAPDH	FW	GGGTCCCAGCTTAGGTTCAT	59.08	55.00	20	248
	RW	CATTCTCGGCCTTGACTGTG	58.92	55.00	20	

Tm: Melting temperature; bp: base pair; FW: forward; RW: reverse.

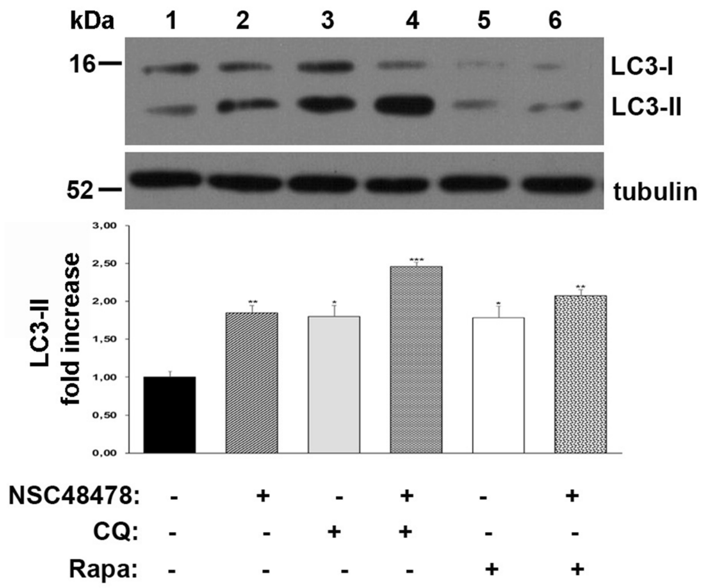

Figure S1. Inhibition of 37/67kDa laminin receptor induces formation of lipidated LC3-II isoform. GT1 cells were grown as in Figure 1, with the exception that here Rapamycin treatment has been shown and LC3-II quantified as in the plot, which shows the fold increase of LC3-II level respect to untreated conditions set as 1 (* $p < 0.05$; ** $p < 0.01$; *** $p < 0.001$).

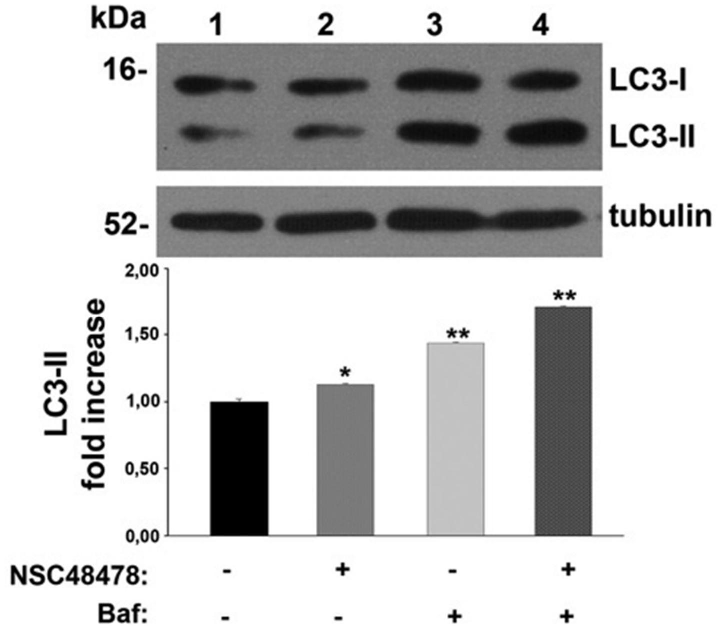

Figure S2. NSC48478 induces formation of lipidated LC3-II isoform. GT1 cells were grown as in Figure 1, with the exception that here Bafilomycin treatment (Baf 100 nM, 24 h) has been shown and LC3-II quantified as in the plot, which shows the fold increase of LC3-II level respect to untreated conditions set as 1 (* $p < 0.05$; ** $p < 0.01$; *** $p < 0.001$).

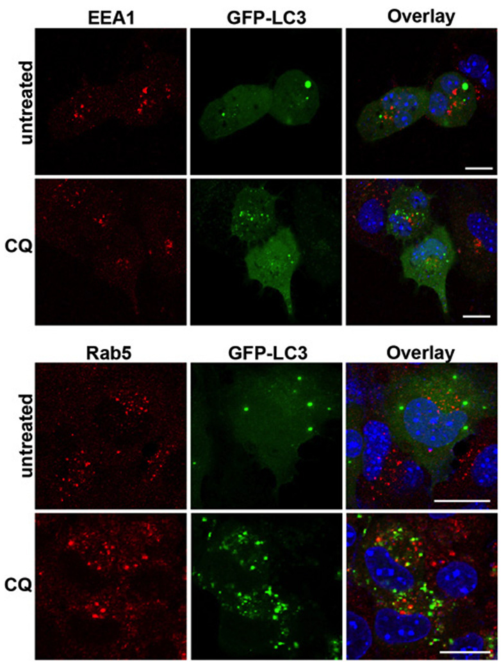

Figure S3. LC3 is not recruited on endosomes under CQ treatment. GFP-LC3 transfected GT1 cells were grown on dishes in 1% serum, and were left untreated, or treated with CQ (50 µM). EEA1 and Rab5 were detected by immunofluorescence analysis. Scale bars: 10 µM.

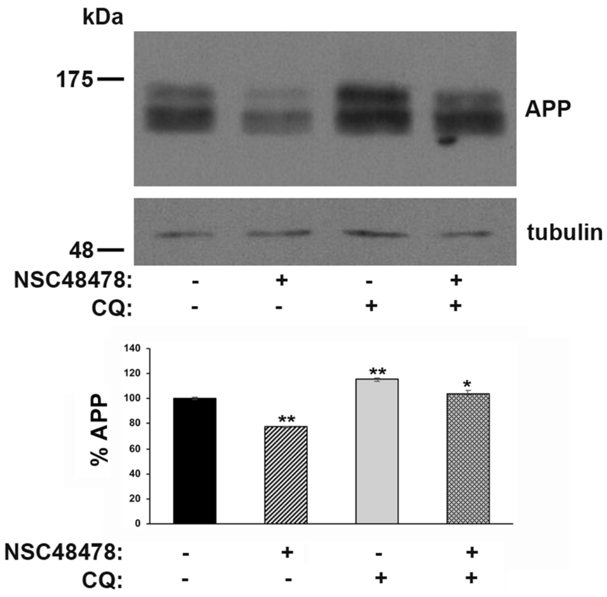

Figure S4. APP levels decrease after NSC48478 administration and increase after the use of CQ. GT1 cells grown on dishes in 1% serum, were left untreated (-) or treated (+) with NSC48478 and/or CQ. APP was revealed by probing the membrane with anti-APP antibody and tubulin was used as loading control. The gels are representative of three independent experiments plotted in the graph, where different densitometric analysis of bands from the gels were compared to untreated conditions, which were set as 100%. Significance is shown with asterisks (* $p < 0.05$; ** $p < 0.01$).

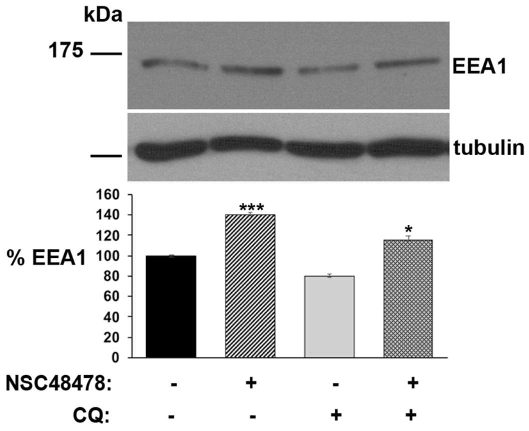

Figure S5. EEA1 levels increase after NSC48478 administration. GT1 cells were grown on dishes in 1% serum and were left untreated (-) or treated (+) with NSC48478 and/or CQ. EEA1 was revealed by probing the membrane with anti-EEA1 antibody and tubulin was used as loading control. Different densitometric analysis of bands from the gels were compared to untreated conditions, which were set as 100%. Significance is shown with asterisks (* $p < 0.05$; *** $p < 0.001$).

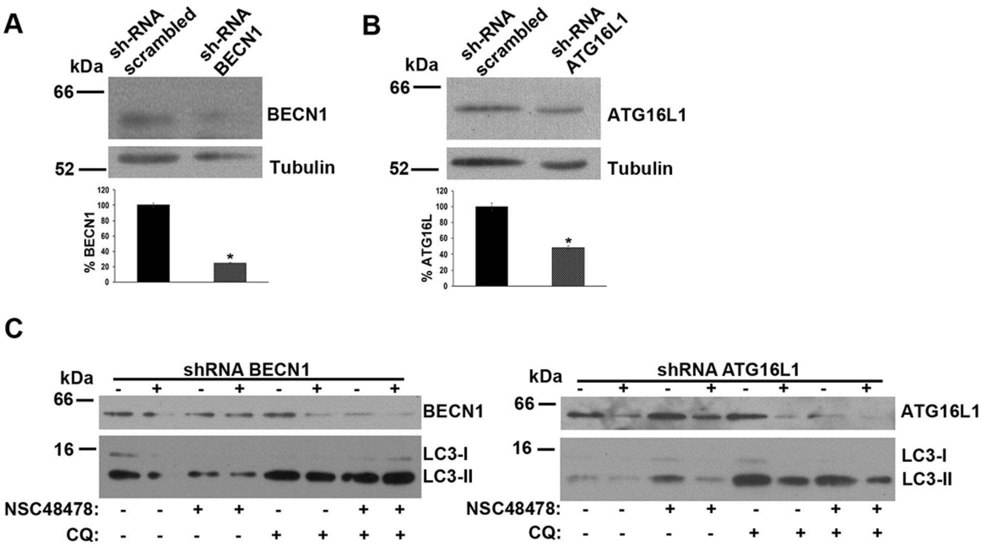

Figure S6. Effects of BECN1 or ATG16L1 downregulation on LC3-II formation after NSC48478 treatment. GT1 cells were transiently transfected with specific shRNAs for BECN1 (**A**) or ATG16L1 (**B**). Levels of ATG proteins were evaluated by western blotting using specific primary antibodies followed by ECL assay. Histograms in the upper panels show reduction of both BECN1 and ATG16L1 after knock down. ShRNA-GFP was used as scrambled (* $p < 0.05$) and tubulin as loading control. (**C**) After NSC48478 and/or CQ administration, levels of lipidated LC3 were evaluated by immublotting the same membrane both with anti-LC3 antibody and anti-BECN1 or anti-ATG16L1, in order to monitor the ATGs knock down level in the same sample.
